# Microbial biodiversity of meadows under different modes of land use: catabolic and genetic fingerprinting

**DOI:** 10.1007/s11274-017-2318-2

**Published:** 2017-07-05

**Authors:** Agnieszka Wolinska, Magdalena Frąc, Karolina Oszust, Anna Szafranek-Nakonieczna, Urszula Zielenkiewicz, Zofia Stępniewska

**Affiliations:** 1 Department of Biochemistry and Environmental Chemistry, The John Paul II Catholic University of Lublin, Konstantynów 1 I Str., 20-708 Lublin, Poland; 2 Institute of Agrophysics, Polish Academy of Sciences, Doświadczalna 4, 20-290 Lublin, Poland; 3 Department of Microbial Biochemistry, Institute of Biochemistry and Biophysics PAS, Pawińskiego 5a Str, 02-206 Warsaw, Poland

**Keywords:** Meadows, Bacterial diversity, CLPP, 454-Pyrosequencing

## Abstract

The main goal of the study was to find differences in the bacterial community structure resulting from different ways of meadow management in order to get the first insight into microbial biodiversity in meadow samples. The next generation sequencing technique (454-pyrosequencing) was accompanied with the community level physiological profiling (CLPP) method in order to acquire combined knowledge of both genetic and catabolic bacterial fingerprinting of two studied meadows (hayland and pasture). Soil samples (FAO: *Mollic Gleysol*) were taken in April 2015 from the surface layer (0–20 cm). Significant differences of the bacterial community structure between the two analyzed meadows resulted from different land mode were evidenced by pyrosequencing and CLPP techniques. It was found that Alpha- and Gammaproteobacteria dominated in the hayland, whereas Delta- and Betaproteobacteria prevailed in the pasture. Additionally, the hayland displayed lower Firmicutes diversity than the pasture. Predominant bacterial taxa: Acidobacteria, together with Chloroflexi and Bacteroidetes seemed to be insensitive to the mode of land use, because their abundance remained at a similar level in the both studied meadows. The CLPP analysis confirmed much faster degradation of the carbon sources by microorganisms from the hayland rather than from the pasture. Amino acids were the most favoured carbon source groups utilized by microorganisms in contrast to carbohydrates, which were utilized to the lowest extent. The study clearly proved that the consequences of even moderate anthropogenic management are always changes in bacterial community structure and their metabolic activity. Bacterial taxa that are sensitive and resistant on modes of land use were determined.

**Electronic supplementary material:**

The online version of this article (doi:10.1007/s11274-017-2318-2) contains supplementary material, which is available to authorized users.

## Introduction

The knowledge of the diversity of microorganisms is essential for a proper and complete understanding of the microbial composition and function of the soil ecosystem (Nannipiperi et al. 2003; Oszust et al. 2014; Gleeson et al. 2016). Awareness of the biodiversity is extremely important because of the role of microorganisms in mineralization processes and in nutrient cycling, which is directly connected with a decline in intensive farming and, in consequence, a decrease in the use of mineral fertilisers and pesticides (Thiele-Bruhn et al. 2012; Oszust et al. 2014; Creamer et al. 2016; Gleeson et al. 2016). Additionally, changes in the microbial community structure are an important element of soil quality monitoring (Frąc et al. 2012; Siczek and Frąc 2012; Creamer et al. 2016; Stone et al. 2016).

The latest approaches for estimation of microbial diversity use direct shotgun next generation sequencing—NGS (*e.g*. Roche 454-pyrosequencing, Illumina). A valuable property of the metagenomic approach is that it provides effective characterization of the genetic diversity present in samples regardless of the availability of laboratory culturing techniques (Roesch et al. 2007; Luo et al. 2012; Tang et al. 2016). In parallel, new technologies facilitating faster and cheaper sequencing now provide unique opportunities to sequence uncultured microbes sampled directly from their habitats, thus expanding and literally transforming our view of the microbial world (Luo et al. 2012).

Estimates of the number of bacterial species in different soils vary between 2000 and 8.3 million (Gans et al. 2005), however, direct analyses by the NGS approaches have indicated lower number of OTUs, ranging from a few dozens and hundreds to thousands and tens of thousands (Chen et al. 2017). Janssen (2006) suggested that 16S rRNA genes from soil bacteria are associated with at least 32 phylum-level groups. The dominant phyla in the known libraries are: Proteobacteria, Acidobacteria, Actinobacteria, Verrucomicrobia, Bacteroidetes, Chloroflexi, Planctomycetes, Gemmatiomonadetes, and Firmicutes (Janssen 2006; Tang et al. 2016). Apart from these nine major phyla, members of a number of other phylum-level lineages, such as: Chlamydiae, Chlorobi, Cyanobacteria, Deinococcus-Thermus, Fibrobacteres, and Nitrospira, can be found in the global data set (Janssen 2006). The dominant phyla across grasslands sites are usually (Will et al. 2010): Acidobacteria (27%), Betaproteobacteria (15.8%), Actinobacteria (11.6%), Gammaproteobacteria (11%), Alphaproteobacteria (9.7%), Deltaproteobacteria (5.1%), Chloroflexi (3.8%), Firmicutes (3.2), and Bacteroidetes (1.45%). Nacke et al. (2011) by pyrosequencing technique found that the dominants in grasslands ecosystem are representatives of: Actinobacteria (16.1%), followed by Acidobacteria (18.7%), then Alpha-(11.4%), and Betaproteobacteria (5.9%). Predomination of Proteobacteria (17.2%) and Acidobacteria (>10%) in alpine grasslands ecosystems, as well as high (>5.0%) abundance of Bacteroidetes, Gemmatimonadetes, and Verrucomicrobia were also reported by Zhang et al. (2014). Similar trend for the main bacterial phyla was confirmed by Leff et al. (2015) as an effect of metagenomic studying of 25 grasslands sites. They also determined the most abundant bacterial phyla in grasslands, that including Proteobacteria, Acidobacteria, Verrucomicrobia, Actinobacteria and Bacteroidetes.

Community level physiological profiling (CLPP) is a cultivation-based technique able to discriminate treatments with bias towards populations, growing under assay conditions (Sharma et al. 1998; Ros et al. 2008). This method is based on the microbial ability for oxidation of different substrates of carbon, and in consequence, it gives information about the metabolic activity of microorganisms, often referred to as bacterial functional diversity (Gomez et al. 2006; Frąc et al. 2014). It is known from the work performed by Haack et al. (1995) and Lehman et al. (1995) that fewer than 95 different substrates are sufficient to evaluate changes in the soil physiological microbial fingerprinting. Consequently, a 96-well microplate with 31 substrates and a control sample in triplicates (EcoPlate) are recommended (Gomez et al. 2006; Islam et al. 2011). The CLPP is regarded to be specific for land use, soil management, and soil texture and reflects some aspects of functional diversity, rather than the community structure (Rutgers et al. 2016).

We combined the CLPP method with the NGS technique in order to gain comprehensive knowledge of both genetic as physiological bacterial fingerprinting of the two studied meadows (hayland and pasture). Therefore, the present work is the first insight into the microbial biodiversity of Polish meadows determined with the newest molecular tools. Our study has great cognitive significance, because the current state of knowledge is fragmentary in the field of microbial biodiversity occurring in meadows, often treated as wastelands. Consequently, the main goal of the study was to determine the differences in the microbial community structure resulting from different modes of meadow management. The metagenomic analyses (NGS, 454-pyrosequencing) were supported by functional microbial diversity assessment (CLPP).

## Materials and methods

### Study site description and soil sampling

The study site was located in the Kosiorów village, the southeast part of Poland—Lubelskie voivodeship (51°13′N; 21°51′E). The climate in this part of the country is moderately warm continental. The long-term annual mean temperature and precipitation at the experimental site are 7.4 °C and 572 mm, respectively (Lipiec et al. 2016).

The two neighbouring agricultural meadows were representative for the site and comprised species-rich grasslands with *Deschampsia cespitosa* L. and *Holcus lanatus* L. as dominant plant species (Wolińska et al. 2013). Importantly, the first meadow was continuously cultivated for haymaking (hayland—coded HAY), fertilized with nitrogen, phosphorus, and potassium in form of Polifoska 8 fertilizer (The Groupa Azoty S.A. Poland) at a dosage recommended by the producer, and mown twice a year in spring and summer (Banach et al. 2009). The second site was used as a control meadow, as it is not mown but grazed (pasture—coded PAS) at a low density of 1 animal/ha (Wolińska et al. 2013) and fertilized once a year (spring) with Saletrzak fertilizer (The Groupa Azoty S.A. Poland). The study performed earlier at the investigated sites by Banach et al. (2009) demonstrated that ammonium-nitrogen is the dominant nitrogen form in the selected areas present at 180 and 279 μmol/kg of soil for PAS and HAY, respectively. Nitrate-nitrogen was in the range 2.20–9.50 μmol/kg of soil for PAS and HAY, whilst phosphorus concentration oscillated between 8.80 (PAS) and 10.60 μmol/kg (HAY). Potassium content ranged from 19.90 mg/kg for PAS to 22.25 mg/kg for HAY.

Soil samples were taken in April 2015 from the surface layer (0–20 cm) with Egner’s bow (three replicates of 0.5 kg, consisting of approximately 50 samples taken from a 100 m^2^ area), strictly according to the sampling rules described in the Polish Norm (PN-R-04031:1997). According to the FAO classification system, the soil investigated was categorized as *Mollic Gleysol*. The samples were stored at 4 °C before processing. The main characteristics of the soil material are presented in Table 1.

**Table 1:** Soils physical and chemical characteristics (±SD)

Land use	Clay (mm)	Silt (mm)	Sand (mm)	Moisture (%)	pH (H_2_0)	Eh (mV)	TOC (%)	BD (Mg/m^3^)
	<0.002	0.002–0.05	0.05–2.0					
HAY	1.31	17.53	81.16	9.10 ± 0.15	5.66 ± 0.03	467.0 ± 0.11	27.4 ± 0.31	0.77 ± 0.05
PAS	1.27	15.09	83.04	7.80 ± 0.09	5.89 ± 0.05	427.4 ± 0.15	21.2 ± 0.27	0.62 ± 0.02

### Soil physical and chemical analysis

Particle size distribution (PSD) was measured using a laser diffractometer Mastersizer 2000 (Malvern, UK) with Hydro G dispersion units (Dobrowolski et al. 2012; Wolińska et al. 2016). The soils were dispersed using ultrasound at 35 W for 4 min without removing the organic matter (Lamorski et al. 2014). The measurements were carried out in three replications. The moisture content was determined according to the ASTM D2216-10 (2011) norm by the gravimetric method (24 h, 105 °C). The acidity (pH) and redox potential (Eh) values were determined from a 2:1 soil suspension in distilled water using a multifunctional potential meter (Hach Lange). The measurements were taken in triplicate after stabilisation of the readings. Bulk density (BD) was determined from a core sample taken by driving a metal corer into the soil. Then, the samples were weighed and BD was calculated from the quotient of the mass of soil solids (Ms) and total soil volume (Vt). Each of the measurements described above was performed in triplicate. Total organic carbon (TOC) was determined in triplicate using an automatic carbon analyser TOC-V_CSH_ SSM 5000A (Shimadzu, Japan).

### DNA extraction procedure and PCR

DNA extraction from soil was performed according the procedure developed by Tomczyk-Żak et al. (2013) with own small modification, regarding purification of the isolate in caesium chloride. Shortly, soil samples (3 g) were mixed with several glass beads (0.4–0.6 mm, Sartorius) in 50 ml Falcon tubes filled with extraction buffer [100 mM Tris–HCl (pH 8.0), 100 mM sodium EDTA (pH 8.0), and 100 mM sodium phosphate (pH 8.0), 1.5 M NaCl] and shaken carefully for a short time. After that, 30 μl of lysozyme (100 mg/ml) was added and the samples were incubated at 37 °C for 30 min. Then, 60 μl of proteinase K solution (20 mg/ml) was added, followed by further incubation at 37 °C for 30 min, after which 1.8 ml of 20% SDS was introduced and the samples were incubated at 65 °C for 2 h and mixed by inversion every 15 min. Subsequently, the samples were centrifuged (10 min, 7000 rpm) at room temperature; the supernatant was guarded, and the sediment was extracted with the same procedure but in half buffer volumes. The two supernatants were combined, extracted with an equal volume of chloroform:isoamyl solution, and precipitated with 0.6 volume of isopropanol. DNA recovered by 20 min centrifugation at 9000 rpm was suspended in 500 μl of water. The crude total DNA was further purified by CsCl gradient centrifugation [16 h, 70,000 rpm, 20 °C; Sorvall WX Ultra (ThermoScietific)]. The concentration and purity of the isolated DNA were assessed with a NanoDrop spectrophotometer (ThermoScientific) after tenfold dilution in triplicate.

### Amplicon preparation and 454-pyrosequencing

Fragments of the correspondent 16S rRNA genes were amplified from soil total DNA using Paq 5000 polymerase (Stratagene) and appropriate primers (27F, 907R) that were fused to Roche-suitable MID oligonucleotides (44 or 33). The PCR conditions were as follows: 95 °C for 3 min, 30 cycles of 95 °C for 30 s, 53 °C for 30 s and 72 °C for 1 min, with a final extension at 72 °C for 7 min. A positive effect of PCR was obtained both for HAY and PAS samples. The PCR product length was 500 bp. Next, the PCR products were purified with a Nucleo Extract II kit (Macherey–Nagel). The concentration and quality of the PCR products were assessed with Pico green staining and a Chip DNA Bioanalyzer, and equal amounts were sequenced using a Roche GS FLX Titanium sequencer with a standard 454 protocol (Illumina, San Diego, CA, USA).

### Analysis of biodiversity

The reads from pyrosequencing were processed using MOTHULITY pipeline (https://github.com/dizak/mothulity, early development stage) which is based upon MOTHUR (Schloss et al. 2009) software and Schloss Standard Operating Procedure described in MOTHUR’s wiki. The sequences were de-noised, filtered based on their quality and aligned using a Silva-compatible database of SSU rRNA genes. Potential chimeras were identified using the Uchime (Edgar et al. 2011) algorithm and subsequently removed. Taxonomic assignments were completed with the RDP classifier (Wang et al. 2007). Operational Taxonomic Units (OTUs) were defined at the threshold of 3% of identity. Rarefaction curves, Krona pie charts and biodiversity indexes were generated with MOTHULITY, based on standard MOTHUR commands output. Oher statistical analyses were performed with STATISTICA 9.0 (StatSoft, USA) software. The normality was checked using the Shapiro–Wilk W test and the homogeneity of variances was assessed by Levene’s test. Microbial community structure were compared to land use mode by using a post hoc Tukey procedure. *p* values of ≤0.05 were considered significant.

### Community level physiological profiling (CLPP)

The Biolog EcoPlate™ system (Biolog Inc., Hayward, CA, USA) was applied for determining the functional fingerprinting of bacterial communities inhabiting HAY and PAS meadows. The 96-well plate consisted of three replicates, with 31 different carbon sources and water blanks (Frąc et al. 2012). Firstly, the soils (1 g) were shaken (20 min, RT) in saline peptone water (99 ml) and incubated (30 min, 4 °C). Secondly, the sample suspension (120 µl) was introduced into each well and incubated (26 °C, 216 h). The tetrazolium violet reduction rate was used to measure the utilization rate (Islam et al. 2011). The data were recorded at 590 nm every 24 h for up to 216 h (Frąc et al. 2014). The data collected at the whole period of EcoPlate™ incubation (0–216 h) were used to calculate the average well colour development (AWCD), substrate richness (*R*), and total carbon source utilization patterns. The biodiversity indices were applied to express the community level physiological profiles of the soil samples as suggested by Gomez et al. (2006).

The AWCD development, *R*, and bacterial OTU number were analysed by ANOVA (Statistica 12.0, StatSoft, USA) and comparisons of means between the treatments were performed using Tukey’s mean separation test at *p* < 0.05.

## Results

### Soil characteristics

The main physical and chemical features of the investigated meadows are shown in Table 1. Taking into account the granulometric composition and the World Reference Base for soil resources (WRB) system, the studied *Mollic Gleysol* was classified as sandy loam, due to the dominance of a coarser fraction (sand). In respect to the moisture level, both sites were characterized by rather law humidity (<10%) and acidic pH. A close relationship between pH and Eh was confirmed in this study. The hayland was characterized by a 20-mV higher Eh level than PAS. The Nernst equation defines that for each of one pH unit, E^0^ becomes more negative by 59 mV. Consequently, for the registered 20 mV disparity in Eh, pH should differ by 0.3 unit, and this is exactly shown in Table 1. The studied meadows differed most strongly in respect to the TOC content, as a 6.2% higher carbon concentration was found in HAY in comparison to PAS. BD amounted to 0.62 and 0.77 Mg/m^3^ for PAS and HAY, respectively, which corresponds to the loose soil category.

### Biodiversity of meadows—the main phyla and classes

**Fig. 1:**
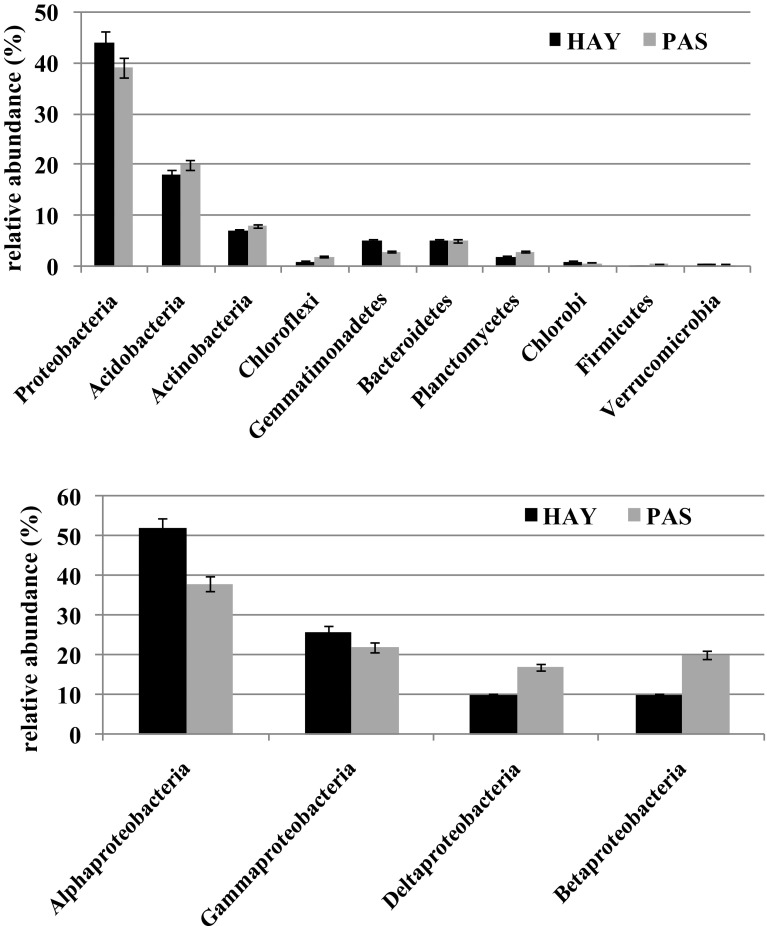
Main phyla of bacteria and Proteobacteria classes detected in *Mollic Gleysol* under different modes of land use. Mean values of three replicates with standard error are presented

The 454-pyrosequencing generated 31 368 and 24 062 sequence reads after quality filtering for PAS and HAY samples, respectively. Unique sequences were clustered into OTU at 0.03 cut-off and assigned to classify the 16S rRNA gene sequences into respective taxonomic branches. All the reads were assigned to Bacteria domain. General diversity statistics for both samples are shown in Table 2. While coverage was high, rarefaction curves calculated at 0.03 dissimilarity level did not reached saturation indicating that much more sequences would be required to capture all the diversity (Fig. 1S, Supplementary material). Both the OTUs number and estimated (ACE, Chao1,* H′*,* 1/D*) species richness reached higher values in PAS community, what indicate on PAS community to be more diverse than HAY.

**Table 2:** Summary of 454-pyrosequencing statistics, species richness and diversity indices at genetic distances of 3% for HAY and PAS sites

Land use	nseqs	Coverage	Chao1	ACE	*H′*	*D*	*1/D*	OTU
HAY	24,062	0.877068	10219.3	13272.5	7.552	0.002163	462.29	5706
PAS	31,368	0.882269	12905.1	16450.7	7.857	0.001296	771.39	7308

*H′* Shannon–Weaver index of general diversity, *D* Simpson index of diversity, *1/D* Simpson index of dominance

The main phyla and classes of bacteria detected with the 454-pyrosequencing technique in respect to the HAY and PAS meadows are presented in Fig. 1. Among all sequences identified in *Mollic Gleysol*, Proteobacteria was found to dominate. Among them, the class of Alphaproteobacteria was the most abundant (52 and 38% of all reads, for HAY and PAS, *p* < 0.05). Representatives of Gammaproteobacteria were noted as the second most abundant microorganisms (26 and 22% of sequences for HAY and PAS, *p* < 0.05). In the third place, classes of Delta- and Betaproteobacteria were found with a similar sequences level (10 and 17–20% for HAY and PAS, *p* < 0.05). Importantly, that mode of land use influenced bacterial diversity. Alpha*-* and Gammaproteobacteria dominated in the HAY site, whereas Delta- and Betaproteobacteria prevailed in the PAS area (Fig. 1).

The second most abundant phylum after Proteobacteria was Acidobacteria (18 and 20% of all reads for HAY and PAS, *p* < 0.05). The abundance of Actinobacteria reached the level of 7–8%, and those bacterial phylum was insensitive to the different land use regimes, like Bacteroidetes representatives (5%). On the contrary, a significant effect of the land management was observed in Gemmatimonadetes and Planctomycetes. Members of the former phylum dominated in the HAY meadow (5%), whereas the latter exhibited the greatest abundance in the PAS site (3%). Sequences classified as Chloroflexi remained on the level of 1–2%, for HAY and PAS, respectively. Other identified phyla, i.e. Chlorobi, Firmicutes and Verrucomicrobia did not exceed 1% of all reads, and remained at a level of 0.3–0.5%, with preference to inhabit the PAS meadow rather than HAY.

### Taxonomic structure of microbial communities across Proteobacteria classes

**Fig. 2:**
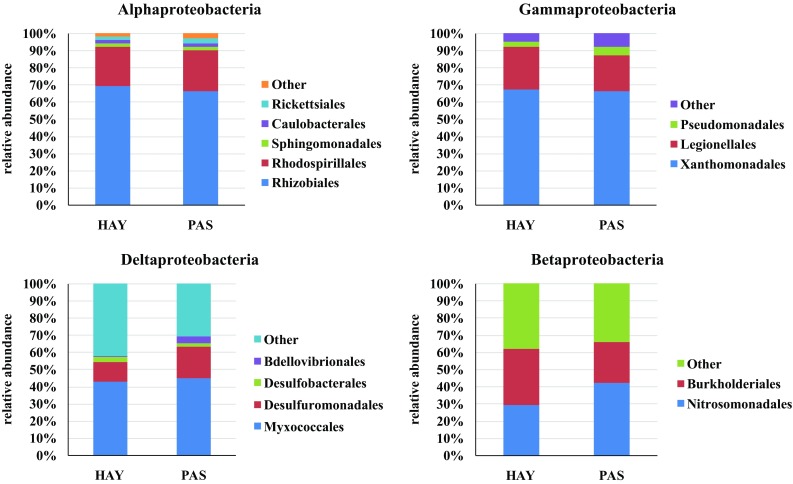
Taxonomic composition based on NGS sequencing across Proteobacteria classes in the two investigated meadows (*HAY* hayland, *PAS* pasture)

Phylogenetic comparison of metagenomes at the level of prevailing orders across Proteobacteria classes is shown in Fig. 2, which also comprises a group of bacteria that were signed as “others”. In this group, sequences of known and identified bacteria with abundance <0.5% are included.

Within Alphaproteobacteria, i.e. the dominant Proteobacteria class in the studied meadows, the most common order was Rhizobiales, which constituted 69 and 66% of sequences for HAY and PAS, respectively (*p* > 0.05). It was followed by Rhodospirillales (24%) which are insensitive to land management, similarly to the two other orders: Sphingomonadales and Caulobacterales (4%). Importantly, the least represented Rickettsiales order (3%) was noted in the PAS meadows only, which might indicate the sensitivity of the members of these order to any human agricultural practices.

The Gammaproteobacteria class was represented by three orders: Xanthomonadales, Legionellales, and Pseudomonadales. The highest abundance was found in Xanthomonadales (67 and 66%, for HAY and PAS, *p* > 0.05); however, the members of the Legionellales order seemed to be the most susceptible to the land use regime (25 and 21%, for HAY and PAS). The smallest group of Gammaproteobacteria was represented by the Pseudomonadales order (3 and 5%), with its preference to inhabit the PAS meadow.

The biodiversity of Deltaproteobacteria was represented by four orders: Myxococcales, Desulfuromonadales, Desulfurobacterales, and Bdellovibrionales. Among them, identified sequences belonging to Myxococcales were the most abundant, ranging from 43% (HAY) to 45% (PAS), *p* > 0.05. They were followed by the order Desulfuromonadales with a higher number in the PAS meadow (18%) rather than in HAY (11%). The two other orders were assigned with bacterial sequences in the range from 1 to 4% (Fig. 2). The abundance of Bdellovibrionales was significantly higher (*p* < 0.05) in the PAS site in comparison to the HAY meadow.

Among Betaproteobacteria, substantially higher sequences number classified to Nitrosomonadales order was found in the PAS meadow (42%) than in HAY (29%). An opposite trend was found in the case of Burkolderiales, which dominated in the HAY area (33%) rather than in the PAS site (24%).

Summarizing, the highest differences in the bacterial community structure in respect to the mode of land use were noted among the Delta- and Betaproteobacteria classes, whereas representatives of Alpha- and Gammaproteobacteria displayed rather strong conservatism in the bacterial structure and seemed to be insensitive to land management practices.

### Taxonomic structure across other phyla detected in HAY and PAS meadows

**Fig. 3:**
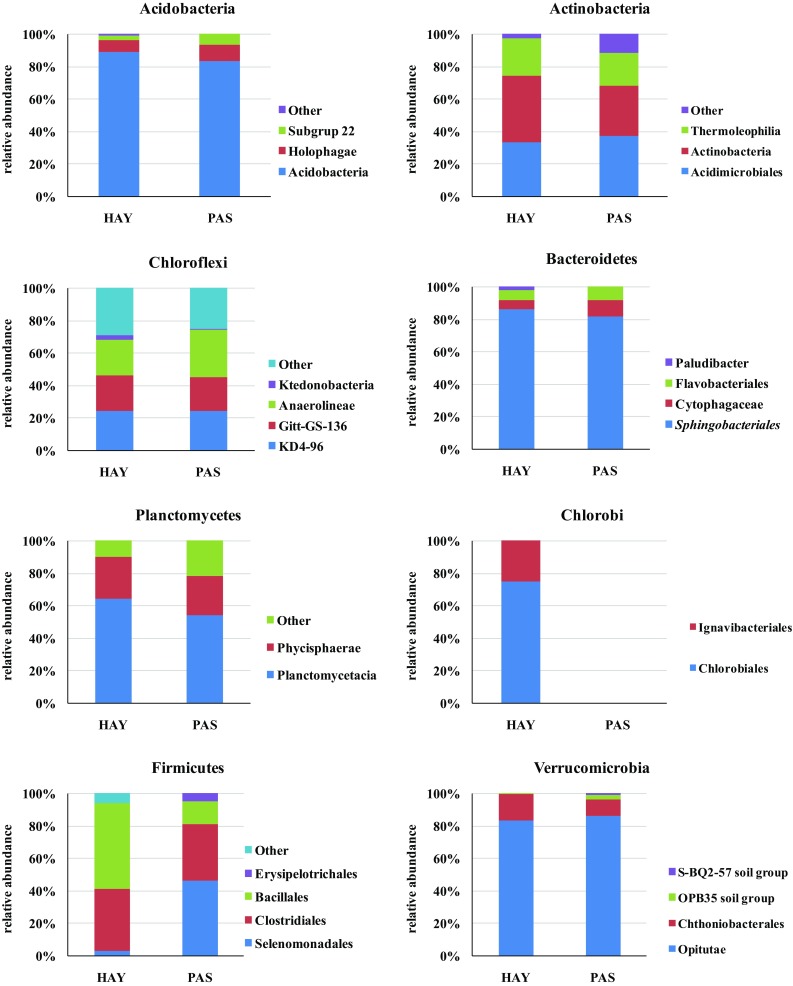
Phylogenetic comparison of soil metagenomes across other phyla detected in the two investigated meadows (*HAY* hayland, *PAS* pasture)

The soil metagenomic structures across other phyla detected in the two studied meadows are shown in Fig. 3. Acidobacteria dominated in HAY (89%) rather than in PAS (83%, *p* < 0.05). Amongst these phyla, abundance of Holophagae was detected, and an increase in the number of its representatives was observed in PAS (10%), in comparison to HAY (7%, *p* < 0.05). The 454-pyrosequencing approach within the Acidobacteria diversity found also subgroup six to be present both in PAS (7%) and HAY (3%, *p* < 0.05).

The Actinobacteria phylum was composed mainly of Acidomicrobiales and Thermoleophilia members. Slight dominance of Acidomicrobiales was found in the PAS meadow (37%) compared with HAY (33%). In contrast, an increase of sequences belonging to Actinobacteria (41%) and Thermoleophili*a* (23%) was evidenced in HAY.

The bacterial community structure of Chloroflexi was similar in the two studied meadows, with two exceptions: (1) the Anaerolineae class, which was more abundant in PAS (29%) rather than in HAY (23%, *p* < 0.05), and (2) sequences of Ktedonobacteria, which were more numerous in the HAY meadow (3%) than in PAS (1%, *p* < 0.05).

In the case of the Bacteroidetes phylum, bacteria from the order of Sphingobacteriales dominated, constituting 76 and 84% of all reads for PAS and HAY, respectively. Members of the Flavobacteriales order and the family Cytophagaceae were subdominants. Both of them preferred the PAS meadow (9%) than HAY (6%). Crucial is the fact that the presence of *Paludibacter* (2%) was confirmed only in the HAY site.

The Planctomycetes composition was represented by Planctomycetacia and Phycisphaerae dominant in the HAY meadow (64 and 26%, respectively). A lower number of their sequences were found in the PAS site, i.e. 24 and 54% for Phycisphaerae and Planctomycetacia, respectively.

Chlorobi representatives were detected in the HAY meadow only; they belonged to two orders: Chlorobiales (75%) and Ignavibacteriales (25%).

High diversification in the bacterial structure between the investigated meadows was found for the Firmicutes phylum. The Firmicutes community was composed of the following orders: Selenomonadales, Clostridiales, Bacillales, and Erysipelotrichales. Importantly, the HAY site was characterized by lower Firmicutes diversity, limited to Bacillales (53%), Clostridiales (38%), and Selenomonadales (3%), in comparison to the PAS meadow. In contrast, the bacterial composition in PAS was shown to be more divergent than in HAY, and distinct dominance of Selenomonadales (46%) was evidenced. Sequences classified to Clostridiales amounted to 35%, whereas those of Bacillales accounted for 14%. Furthermore, the presence of Erysipelotrichales (5%) was confirmed only in the PAS meadow. Finally, the Verrucomicrobia phylum was dominated by bacteria from the class of Opitutae (83 and 86% for HAY and PAS, respectively). Then, members of Chthoniobacterales were noted (10 and 16% for PAS and HAY, respectively). Other bacteria belonged to the OPB 35 and S-BQ2-57 soil groups, but their relative abundance did not exceed 3%. The Gemmatimonadetes class shown in Fig. 2 is not included in Fig. 3 because there were no differences in the composition of microorganisms between the HAY and PAS meadows. In both cases, 98% of sequences belonged to Gemmatimonadetes, whilst 2% were included in “others”.

Performed beta-diversity analysis (Fig. 2S, Supplementary material) confirmed those of alpha-diversity findings (Table 2) and demonstrated higher number of species (7308) in PAS site than in HAY (5706). Total richness for all groups amounted 10,931. The community overlap, depicted as Venn diagram (Fig. 2S) showed 19% of shared OTUs clustered at the 0.03 level. The shared OTUs belonged mainly to….

### Catabolic activity in HAY and PAS meadows

The data obtained from 454-pyrosequencing were complemented by the CLPP analysis in order to deepen the knowledge of bacterial catabolic activity, functional diversity, and preference to use different carbon sources located on Biolog EcoPlate™. The Standard Biolog EcoPlate™ system contains carbon substrates belonging to five groups: carbohydrates (CH), carboxylic and acetic acids (CA), amines and amides (AD), amino acids (AA), and polymers (P).

**Fig. 4:**
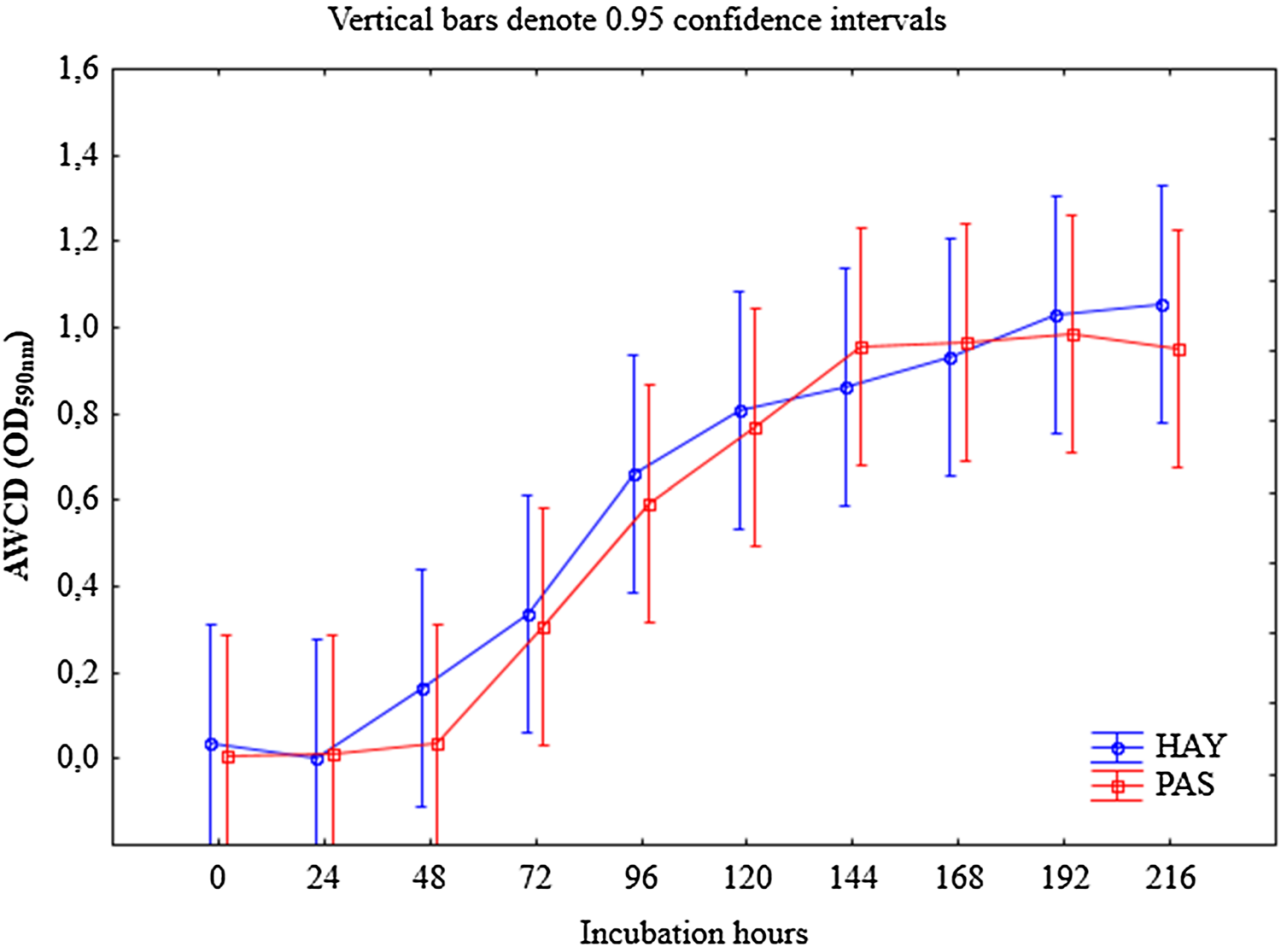
Average well-colour development (AWCD) of metabolized substrates calculated from Biolog EcoPlate™ data for the two studied meadows: hayland (HAY) and pasture (PAS). *Vertical bars* represent the 0.95 confidence intervals

As illustrated in Fig. 4, the microbial activity expressed as average well colour development values (AWCD), displayed an increasing trend with incubation time; however, it did not vary significantly between the HAY and PAS meadows (*p* > 0.05), which may suggest that the microbial communities are stable in both sites. Until 48 h of the incubation period, there was no substrate utilization by microorganisms. However, since 72 h to the end of the experiment (216 h), an increase in the AWCD values was noted for both meadows, with slight predominance for HAY (*p* > 0.05).

**Fig. 5:**
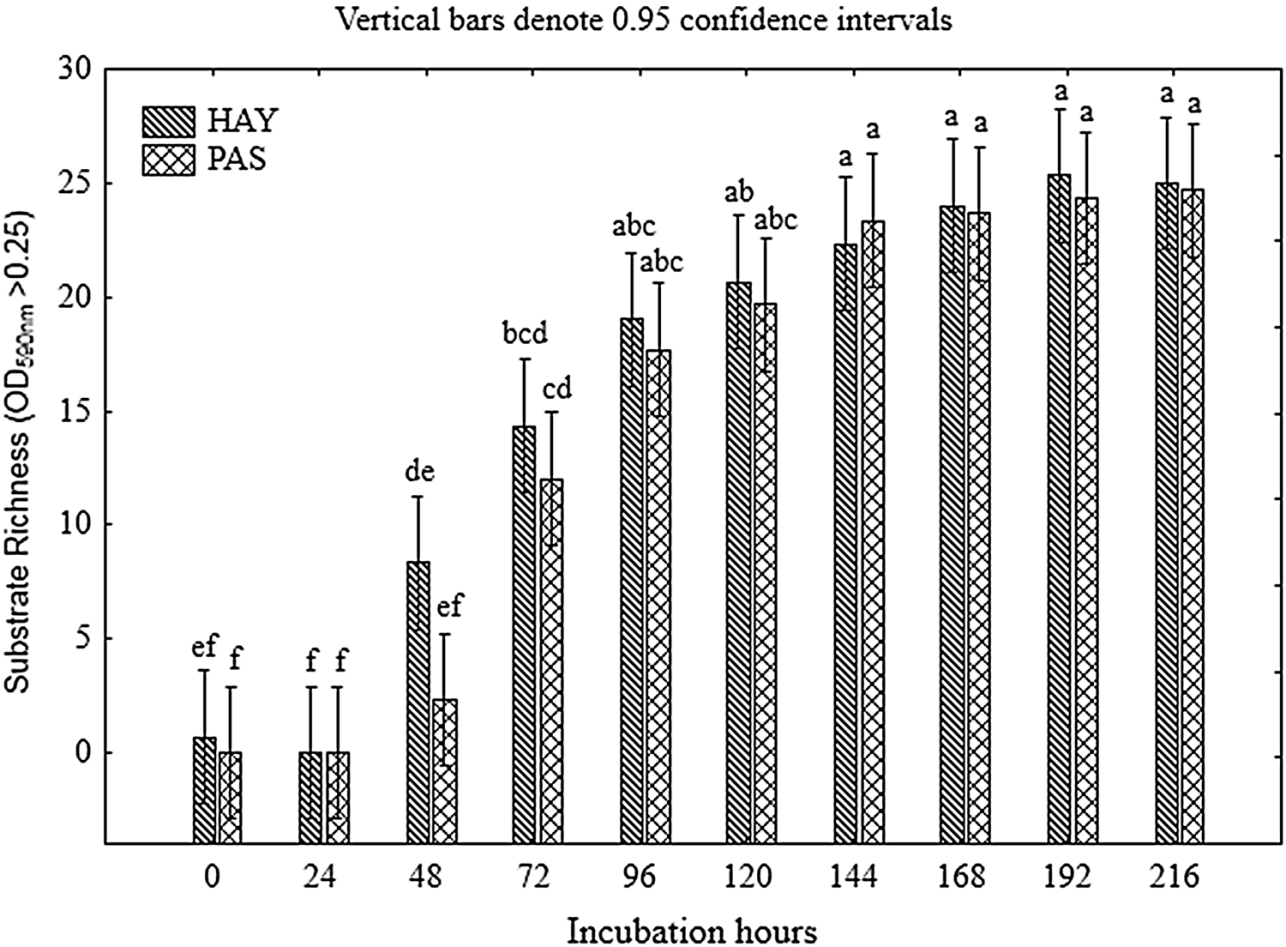
Substrate richness of metabolized substrates calculated from Biolog EcoPlate™ data for the two studied meadows: hayland (HAY) and pasture (PAS). *Vertical bars* represent the 0.95 confidence intervals. The *different letters* indicate significant differences between the meadows (Tukey’s mean separation test, *p* < 0.05)

The richness index (*R*) displayed a similar trend to that for the AWCD changes (Fig. 5). Significant differentiation in the substrate richness between HAY and PAS was noted at 48 h of incubation (*p* < 0.05). Afterwards, in spite of the slightly higher values of *R* in the HAY meadow, the observed changes were not significant (*p* > 0.05) until the end of the experiment (216 h), as visible in Fig. 5. Soil microorganisms utilized from 0 to 24 of carbon substrates among the 31 compounds tested. Considerably faster degradation of the carbon sources was carried out by the microbial community from the HAY meadow, rather than from PAS. The HAY community utilized 7 carbon compounds at 48 h and 14 carbon compounds at 72 h, whereas the microorganisms from PAS metabolized 2 and 11 substrates, respectively, at the same time. The utilization of the carbon substrates was stabilized at 120–144 h of incubation. The differences in microbial communities’ preference for the various carbon sources were confirmed by the differences in the microbial structure between HAY and PAS described above.

**Fig. 6:**
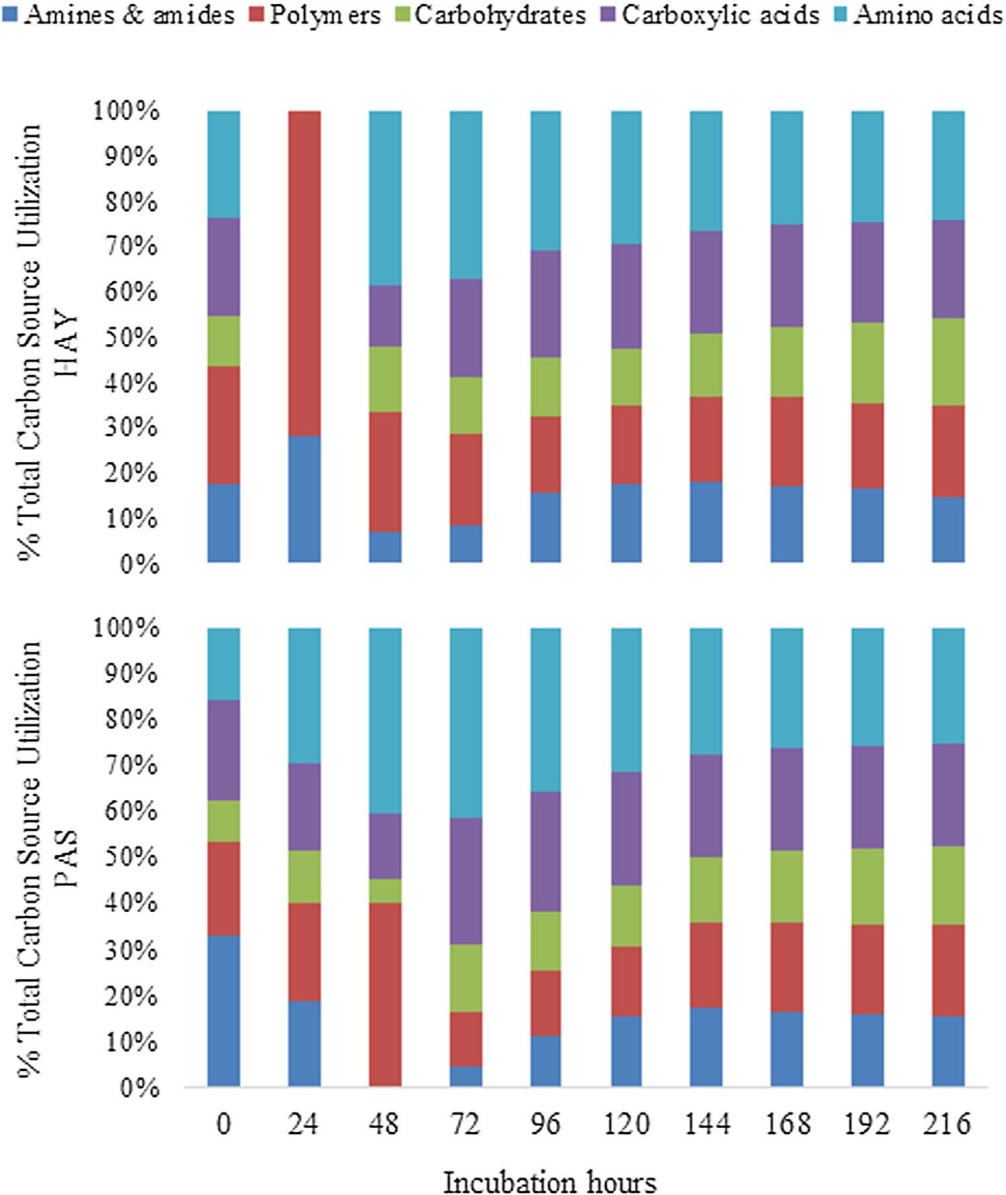
Total carbon source utilization response (%) tracked according to the meadow type (HAY-hayland and PAS-pasture) for the different carbon substrate groups

**Fig. 7:**
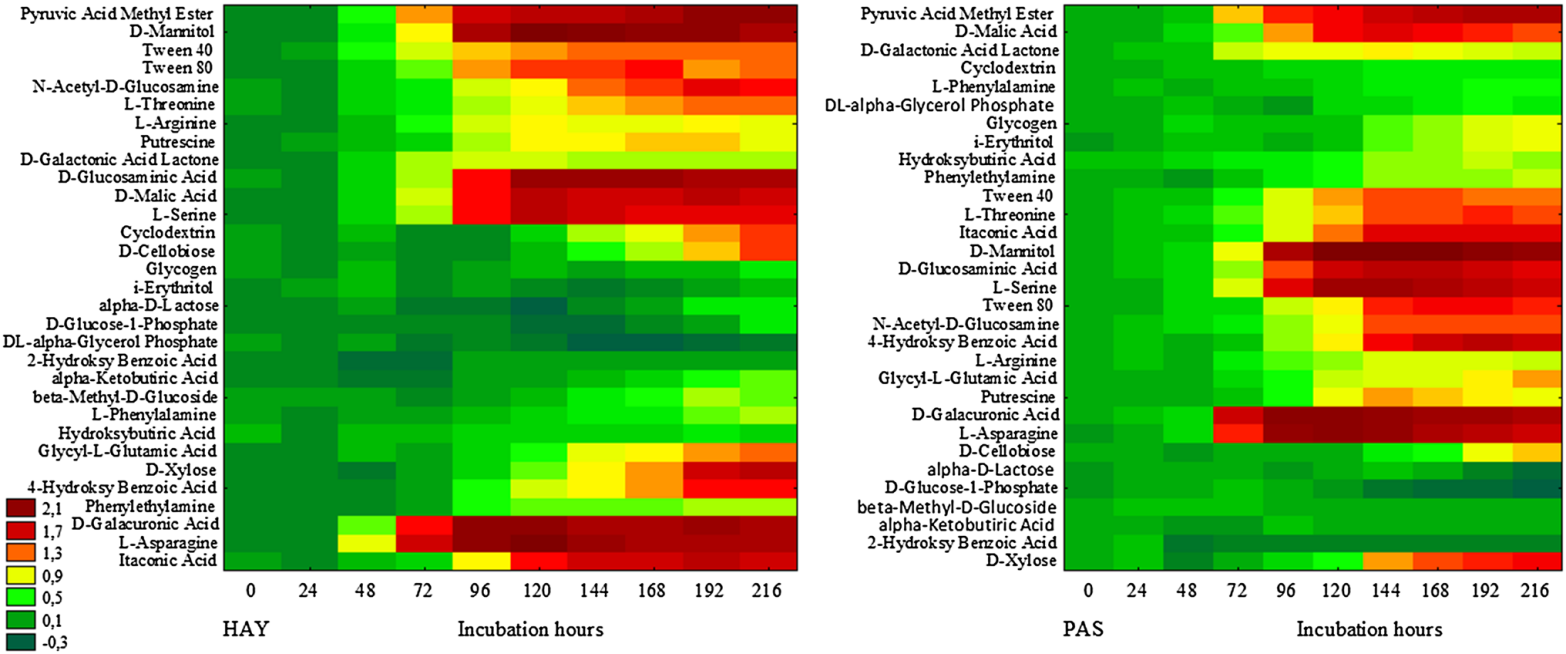
Biolog EcoPlate™ diagram of the intensity of carbon substrate utilization. *HAY* hayland, *PAS* pasture, n = 3

At the beginning of the experiment (0 h), microorganisms inhabiting the HAY meadow started to utilize AA (25%), P (23%), and CA (21%), Fig. 6. At the same time, those colonising the PAS site preferred AD (32%), CA (22%), and P (21%). The most visible differences in the metabolic activity between the two studied meadows were registered at 24 h of incubation. In the case of HAY, the microorganisms metabolized P (72%) and AD (28), whereas those from PAS most of all utilized AA (30%) and P (21%). Since 48 h of incubation, an increase in AA utilization was noted in both meadows, with dominance in the PAS site. Variations were also observed in respect to polymers; their utilization reached 30–40% for PAS and HAY. The patterns of carbon substrate utilization by the microorganisms started to be similar in the two meadows since 96 h of the experiment. Starting from this time (96 h) to the end of the incubation, the utilization of the carbon sources was as follows: AA > CA > P > AD > CH (Fig. 3S, Supplementary material). Generally, throughout the study period, carbohydrates were at the least preferred carbon group for the HAY and PAS microbial communities, in contrast to the most favoured C-source group, i.e. amino acids (Fig. 3S). The observations mentioned above were additionally confirmed by the diagram of the intensity of carbon substrate utilization, as shown in Fig. 7. These heat maps of categorized substrate utilization patterns of the microbial communities from the HAY and PAS meadows during 0–216 h of incubation clearly revealed differences in microbial activity between the two meadows studied. Intensive utilization of carbon substrates began to increase since 72 h of incubation. At the same time, microbial communities from HAY and PAS showed similar metabolic behaviour and primarily preferred to utilize l-asparagine, d-galacturonic acid, and pyruvic acid methyl ester. The next substrates utilized most intensively by the HAY community were l-asparagine, d-galacturonic acid, d-glucosaminic acid, d-mannitol, d-malic acid, l-serine, pyruvic acid methyl ester, and tween 80. However, since 192 h of the experiment, the HAY inhabitants also intensively metabolized d-xylose, 4-hydroksybenzoic acid, and *N*-acetyl-d-glucosamine. In the PAS meadow community, carbon substrates that were most intensively utilized from 77 h included the following: l-asparagine, d-galacturonic acid, d-mannitol, l-serine, pyruvic acid methyl ester, and d-malic acid. Subsequently, since 144 h of incubation, other carbon sources started to be metabolized, i.e. itaconic acid, d-glucosaminic acid, tween 80, 4-hydroksybenzoic acid, l-threonine, and d-xylose.

To compare more specifically the metabolic activity of HAY and PAS, the most concise heat map was constructed (Fig. 4S, Supplementary material). Based on this comparison, it was demonstrated that the utilization of l-asparagine, pyruvic acid methyl ester, and d-glucosaminic acid was more intensive in the HAY site than in PAS. In contrast, the microbial community from the PAS meadow more effectively metabolized d-mannitol, d-galacturonic acid, and l-serine, in comparison to microorganisms from the HAY site (Fig. 4S).

## Discussion

The effect of different modes of land use on microbial catabolic activity and community structure was examined in two meadows (HAY, PAS). It should be emphasized that grassland communities are threatened and in retreat not only in Poland but also everywhere in Europe (Van der Hoek and Sykora 2006; Kącki and Michalska-Hejduk 2010; Bucher et al. 2016).

At the beginning of the study, the physical and chemical soil characteristics were determined in order to find the differences in soil chemistry between the studied meadows. It is commonly known that soil conditions (both physical as chemical) are strong determinants of the composition of the soil microbial community (Hamarashid et al. 2001; Banach et al. 2009; Neuenkamp et al. 2013; Wolińska et al. 2013; Bucher et al. 2016). PSD is one of the most important soil parameters. It plays a crucial role in carbon storage and influences nutrient availability (Hamarashid et al. 2001), and thus is a critical factor for soil microbial activity (Thiele-Bruhn et al. 2012; Wolińska et al. 2016). The investigated *Mollic Gleysol* seemed to belong to the sandy loam category. The nutrient level (N and P) and chemical parameters (i.e. organic matter, total S, Fe, and Ca contents) in both meadow types were described by Banach et al. (2009). According to Neuenkamp et al. (2013), hay removal is known to remove nutrients. This is however contrary to our earlier observations (Banach et al. 2009), as higher levels of nitrates and phosphorus were detected in the HAY meadow rather than in PAS. The nitrate content amounted to 9.46 and 2.24 µmol/kg whilst total P was 10.51 and 8.83 µmol/kg for HAY and PAS, respectively (Banach et al. 2009). This trend indicates fertilization practices (twice a year) in the HAY meadow. Nevertheless, the small differences in the P content between the HAY and PAS locations may suggest that PAS had also been fertilized in the past (Banach et al. 2009). Microorganisms usually prefer those soils for colonization that are non-compacted and characterized by good oxygenation status (Wolińska et al. 2013). These terms and conditions were fulfilled by both the HAY and PAS meadows (Table 1), with BD values 0.62–0.77 Mg/m^3^ and Eh above 400 mV. Therefore, being aware of the fact that the soil conditions are conducive to the microbial growth, we decided to explore the differences in their community structure (genetic fingerprinting) and metabolic activity (physiological fingerprinting) depending on the land use regime.

It is worth to mention, that soil microbial community is composed of both prokaryotes, eukaryotes (fungi, algae), archaea, protozoa and viruses. Each of them contribute in soil biological activity. However, the 16S rRNA primers (27F, 907R) applied for NGS analysis in the current study are universal only for prokaryotes. Simultaneously, 16S-rRNA sequencing has represented a fundamental step for bacteria identification and includes an essential information necessary for their classification (Rosselli et al. 2016). Prokaryotes are absolute dominants in the soil environment in terms of both count (10^8^–10^10^ per 1 g of soil) and species diversity (Roesch et al. 2007). Archaea abundance is usually estimated for c.a. 10% of prokaryotic species, whilst fungi number equalled c.a. 10^5^–10^6^ per g of soil (Ogunmwonyi et al. 2008). The number of algae and protozoa per soil gram remain on the level of 10^4^–10^5^ (Patova and Dorokhova 2008). Taking into account the domination of prokaryotes in the whole microorganisms structure and the fact that bacteria respond more strongly to land use mode, compared with other microbial groups (Esperschütz et al. 2007), our NGS analysis were concentrated on those microbial group.

The available literature shows that the following bacterial taxons in the phylum range and proteobacterial classes are common in grassland sites (Nacke et al. 2011): Actinobacteria (19.6%), Acidobacteria (18.7%), Alphaproteobateria (11.4%) and Betaproteobacteria (5.9%). The other (less common) types are: Bacteroidetes, Chloroflexi, Verrucomicrobia, Cyanobacteria, Spirochaetes, Gemmatimonadetes, Planctomyces (Will et al. 2010; Nacke et al. 2011). The bacterial community of Polish meadows was predominantly composed of Proteobacteria, constituting 39–44% of all detected sequences. In the HAY meadow, the dominance of Alpha- and Gammaproteobacteria representatives was detected, whereas a majority of microorganisms in PAS represented the classes of Delta- and Betaproteobacteria (Fig. 1). Importantly, the last two classes mentioned showed the greatest diversity in terms of microbial structure (Fig. 2). These findings are in agreement with Zhang et al. (2014) observations performed for alpine grasslands ecosystems, and with Will et al. (2010) study. The second phylum in terms of the number of sequences was Acidobacteria that remains at a similar level (20%) to those reported by Naether et al. (2012) for grasslands. Based on an analysis of 16S rRNA gene clone libraries, Naether et al. (2012) revealed that grassland soils were dominated by subgroup Gp6 and forest soils by subgroup Gp1 Acidobacteria. Subgroup Gp6 was also detected in our study (Fig. 3) and its dominance in the PAS meadow was confirmed. This remains in agreement with the result reported by Naether et al. (2012) within the context of grasslands. Will et al. (2010) also noted that members of Acidobacteria phylum are predominant (c.a. 20%) across nine investigated German grasslands. Moreover, it was emphasized that Acidobacteria appear to play an important ecological role in the grasslands ecosystems functioning (Will et al. 2010). The differences in microbial community structure between agricultural soils and meadows were also observable for the other bacterial phyla. In the meadows, a decrease was found in the level of Actinobacteria sequences (~80%), Firmicutes and Verrucomicrobia (~90%) in comparison to agricultural soils (Hartmann and Widmer 2006). The meadows were richer than the arable soils taking into account the abundance of Chloroflexi (7%), Bacteroidetes, Planctomycetes, and Gemmatimonadetes (5%). Importantly, the dominance of Chloroflexi, Planctomycetes, Firmicutes, and Verrucomicrobia was noted in the PAS meadows, in comparison to the HAY site (Fig. 1). On the contrary, Proteobacteria and Gemmmatimonadetes dominated in HAY rather than in PAS.

The differences in the microbial genetic diversity between the HAY and PAS meadows were also confirmed by their catabolic activity. CLPP analysis allowed recognition of microbial preferences for the intensity of carbon substrate utilization. It was demonstrated that microorganisms from the HAY site degraded carbon substrates much faster than those in PAS (Fig. 5). The calculated effectiveness in C utilization in the case of HAY was 22.5 and 37.8% after 48 and 72 h of incubation, respectively. Simultaneously, the efficiency of PAS microorganisms in carbon sources utilization ranged from 6.45 to 35.5%. The changes in the utilization patterns of the categorized substrates among the microbial communities in the studied treatments suggest that the incorporation of even moderate agricultural activity (as it was in the HAY site) may be enough to cause differences in microbial metabolism. It was also found that, both in the HAY and in PAS meadows, amino acids (AA) were the most preferable source of carbon, whilst carbohydrates (CH) were utilized the least likely. Ros et al. (2006) recommended using the CLPP technique accompanied with other methods (i.e. structural diversity study) when examining the impact of land use on soil biological characteristics. A similar suggestion was proposed by Oszust et al. (2014). Despite the potential limitations of using Biolog EcoPlates™ connected with the fact that this method concerns microorganisms that are cultured on plates and may fail in the case of those uncultured in laboratory conditions (Zak et al. 1994), our results may be supported by the findings reported by Säwström et al. (2016) regarding subsurface seagrass sediments in the Mediterranean Sea. They noted faster carbon substrate utilization in a sandy coastal meadow than in a muddy one. According to Gomez et al. (2006) and Frąc et al. (2016), the carbon sources in the Biolog™ assay provide a wide set of compounds that can be used to estimate the relative potential catabolic versatility. Therefore, in the present study, CLPP was combined with the NGS method.

## Conclusions

The results of our study revealed that there are differences in both genetic and catabolic prokaryotes diversity in meadows and they depend on the mode of land use. The 454-pyrosequencing and CLPP techniques combined together harmonized with each other, and allowed us to distinguish microbial and catabolic differences between the two seemingly similar meadows. Both the OTUs number and species richness indices reached higher values in PAS community, what indicate on PAS community to be more diverse than HAY.

Dominance of Proteobacteria was found in both studied sites. The culture independent technique (454-pyrosequencing) revealed that Alpha- and Gammaproteobacteria dominated in the hayland (HAY), whereas Delta- and Betaproteobacteria prevailed in the pasture (PAS). The greatest differences in the bacterial community structure in respect to the land use regime were noted among Delta- and Betaproteobacteria classes, whereas representatives of Alpha- and Gammaproteobacteria displayed rather strong conservatism in the bacterial structure and seemed to be insensitive to land management practices. Acidobacteria, together with Chloroflexi and Bacteroidetes seemed to be insensitive to the mode of land use, and their abundance was at a similar level in the HAY and PAS sites. In contrast, high diversification in the bacterial structure between investigated meadows was found for the Firmicutes phylum. The HAY displayed lower Firmicutes diversity than the PAS.

The differences in the microbial genetic diversity between the HAY and PAS meadows were also confirmed by the analysis of community level physiological profiling. Much faster degradation of the carbon sources was carried out by microorganisms from the HAY meadow rather than from PAS. The gradation of the carbon source use was as follows: amino acids > carboxylic and acetic acids > polymers > amines and amides > carbohydrates. By combination of the CLPP method with the NGS technique, new knowledge of genetic and functional bacterial fingerprinting of meadows (hayland and pasture) was acquired. The present study is the first to provide insight into the microbial biodiversity of Polish meadows under different management practices determined with the newest molecular tools.

## Electronic supplementary material

Below is the link to the electronic supplementary material.

Supplementary material 1 (DOCX 277 KB)
