# Supplementary material for: Microbial biodiversity of meadows under different modes of land use: catabolic and genetic fingerprinting
Source: World J Microbiol Biotechnol. 2017 Jul 5;33(8):154. doi: 10.1007/s11274-017-2318-2 (PMC5498651; doi:10.1007/s11274-017-2318-2)
Supplement: Supplementary file 1 — Supplementary material 1 (DOCX 277 KB) [file 11274_2017_2318_MOESM1_ESM.docx]

**SUPPLEMENTARY MATERIAL**

**
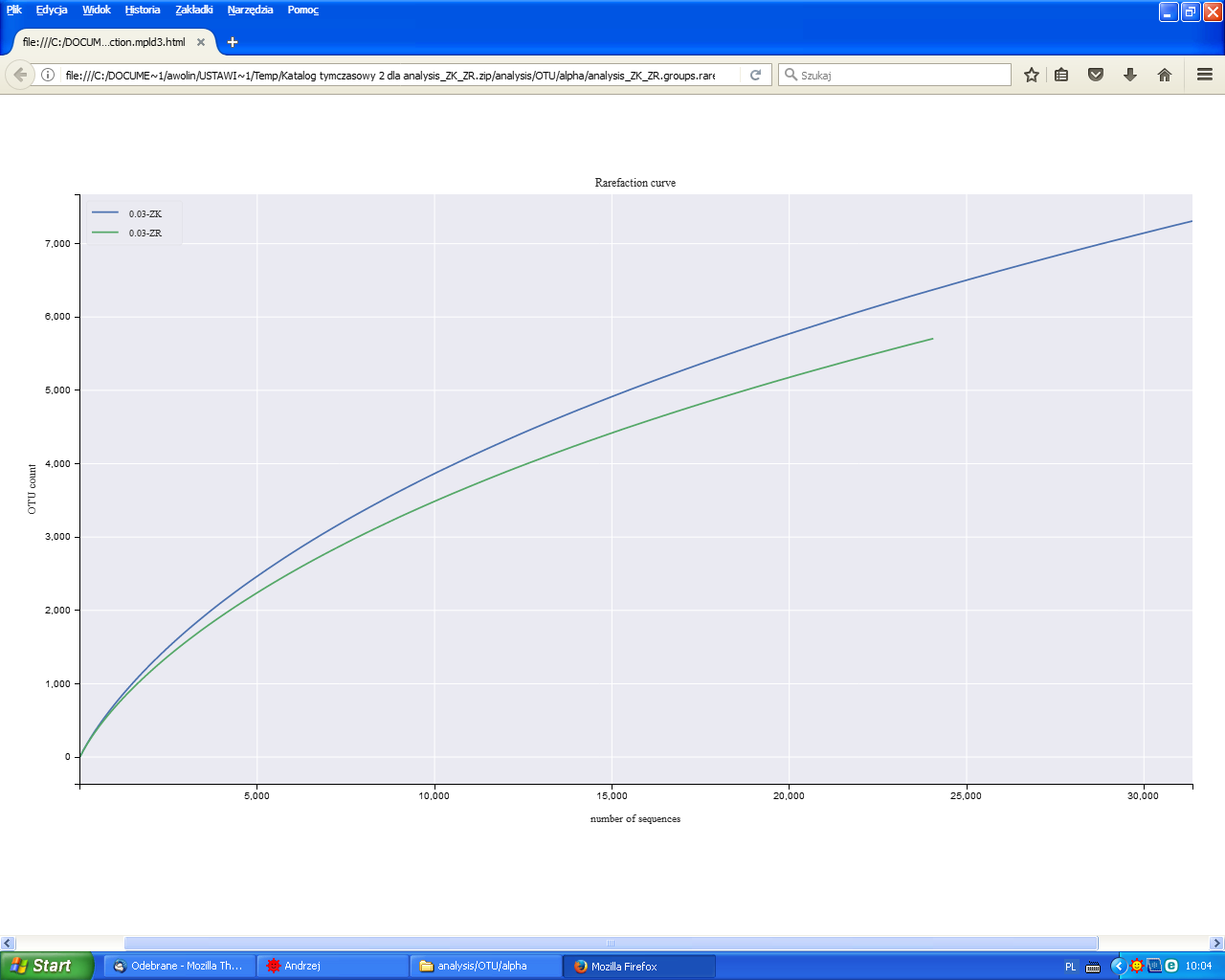
**

**HAY**

**PAS**

**Fig. 1S** Rarefaction curves for the observed OTUs in PAS and HAY meadows, at a genetic distance of 0.03.

**
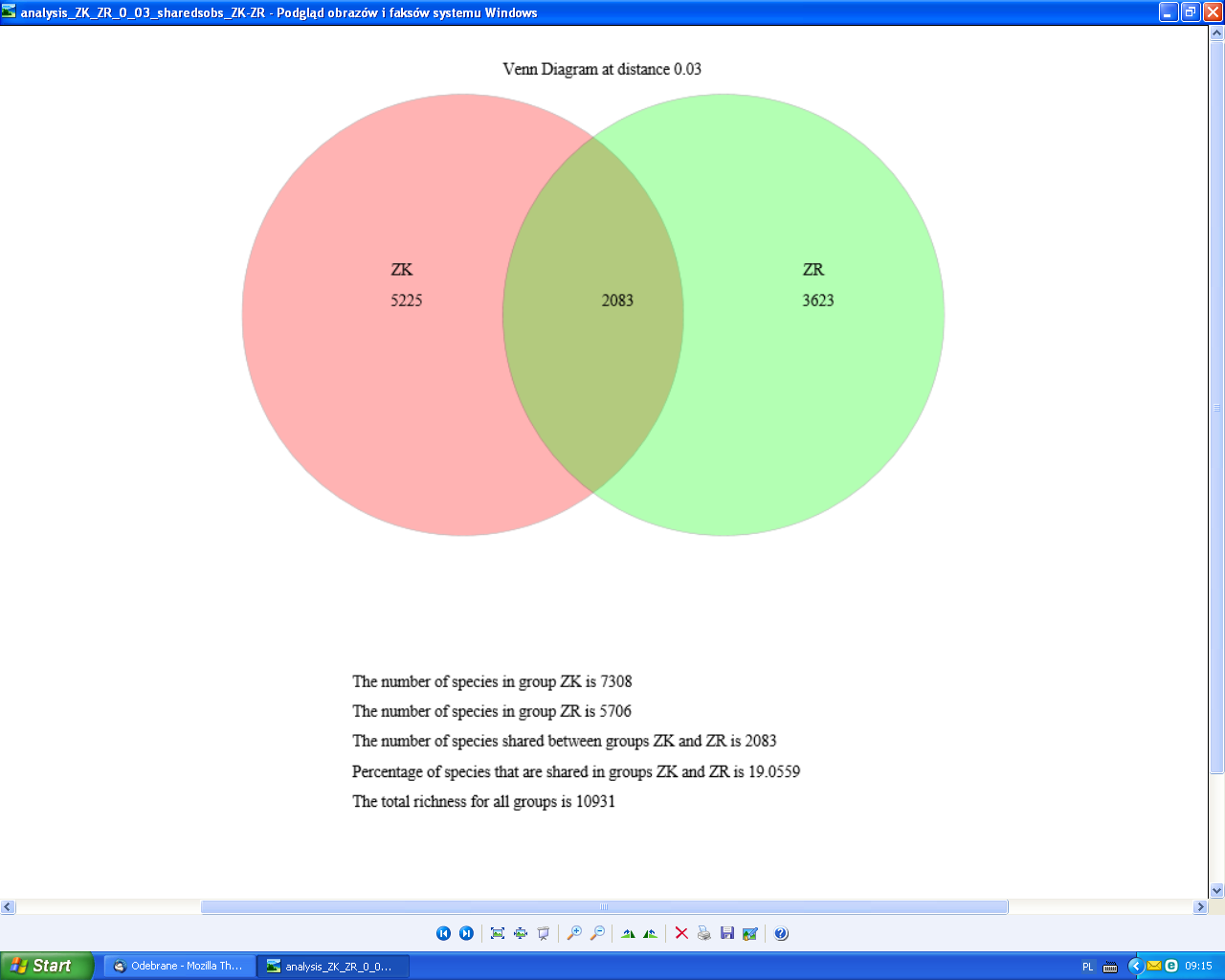
**

**HAY**

**PAS**

**Fig. 2S** Beta-biodiversity in HAY and PAS samples illustrated by Venn diagram.


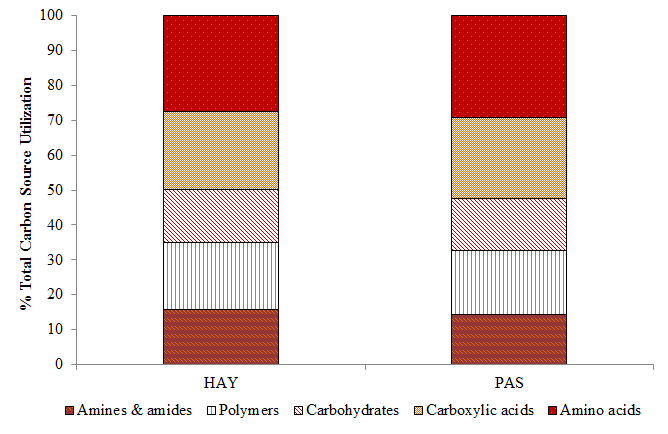


**Fig. 3S** Categorized carbon substrate utilization patterns (%) by the microbial communities from HAY and PAS meadows, respectively (an average of all the experiment time)


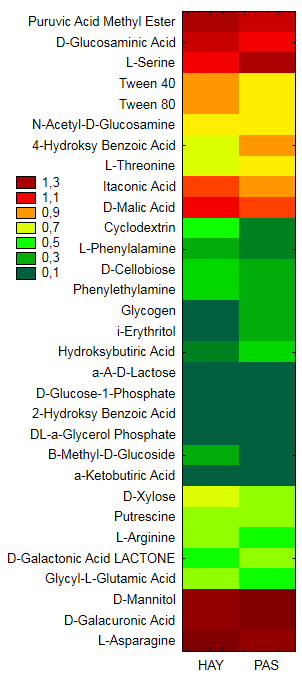


**Fig. 4S** Heat map of categorized substrate utilization patterns by the microbial communities from the HAY and PAS (an average of all the experiment time)
